# Quantitative Analysis and Differential Evaluation of Radix Bupleuri Cultivated in Different Regions Based on HPLC-MS and GC-MS Combined with Multivariate Statistical Analysis

**DOI:** 10.3390/molecules27154830

**Published:** 2022-07-28

**Authors:** Zhenhuan Wang, Huanxi Zhao, Lu Tian, Mengya Zhao, Yusheng Xiao, Shuying Liu, Yang Xiu

**Affiliations:** Jilin Ginseng Academy, Changchun University of Chinese Medicine, Changchun 130117, China; wangzh@ccucm.edu.cn (Z.W.); phoenix8713@sina.com (H.Z.); tianlulu9903@163.com (L.T.); zhaomy@ccucm.edu.cn (M.Z.); xiaoys@ccucm.edu.cn (Y.X.)

**Keywords:** Radix Bupleuri, HPLC-MS, GC-MS, multivariate statistical analysis, saikosaponins, volatile compounds, cultivation region

## Abstract

The quality of Radix Bupleuri is greatly affected by its growing environment. In this study, Radix Bupleuri samples that were harvested from seven different regions across northwest China were examined by high-performance liquid chromatography (HPLC) and gas chromatography (GC) coupled with mass spectrometry (MS) to reveal significant differences in quality contributed by the cultivation region. An HPLC-MS method was firstly established and used in the multiple reaction monitoring mode for the quantitative analysis of five saikosaponins in Radix Bupleuri so as to evaluate the difference in the absolute content of saikosaponins attributable to the cultivation region. The effect on the components of Radix Bupleuri was further investigated based on the profiles of the representative saponins and volatile compounds, which were extracted from the Radix Bupleuri samples and analyzed by HPLC-MS and GC-MS. Multivariate statistical analysis was employed to differentiate the Radix Bupleuri samples cultivated in different regions and to discover the differential compositions. The developed quantitative method was validated to be accurate, stable, sensitive, and repeatable for the determination of five saikosaponins. Further statistical tests revealed that the collected Radix Bupleuri samples were distinctly different from each other in terms of both saponins and volatile compounds, based on the provinces where they were grown. In addition, twenty-eight saponins and fifty-eight volatile compounds were identified as the differentially accumulated compositions that contributed to the discrimination of the Radix Bupleuri samples. The Radix Bupleuri samples grown in Shouyang county showed the highest content of saikosaponins. All of the results indicated that the cultivation region significantly affected the accumulation and diversity of the main chemical components of Radix Bupleuri. The findings of this research provide insights into the effect of the cultivation region on the quality of Radix Bupleuri and the differentiation of Radix Bupleuri cultivated in different regions based on the use of HPLC-MS and GC-MS combined with multivariate statistical analysis.

## 1. Introduction

Radix Bupleuri, which is the dry root of *Bupleurum chinense* DC and narrow leaf *Bupleurum scorzonerifolium* Willd, is a commonly used traditional Chinese medicine. It was firstly recorded in Shennong Traditional Herbal Scriptures and has been widely used in the treatment of inflammation, malaria, influenza, fever, menstrual disorders, and hepatitis for thousands of years [1]. Radix Bupleuri is mainly cultivated in Shanxi, Shaanxi, Hebei, Gansu, and Ningxia provinces in northwest China, because it prefers dry environments and large temperature differences between day and night [2]. The Radix Bupleuri medicinal materials cultivated in China are not only supplied to the domestic market, but also exported to Japan and other countries. The growth of Radix Bupleuri is greatly affected by precipitation, climate, and soil, which lead to different kinds and contents of chemical components in Radix Bupleuri. Therefore, a comparative analysis for differentiating Radix Bupleuri from various main cultivation regions is important and valuable for its quality control and evaluation [3,4,5].

**Figure 1:**
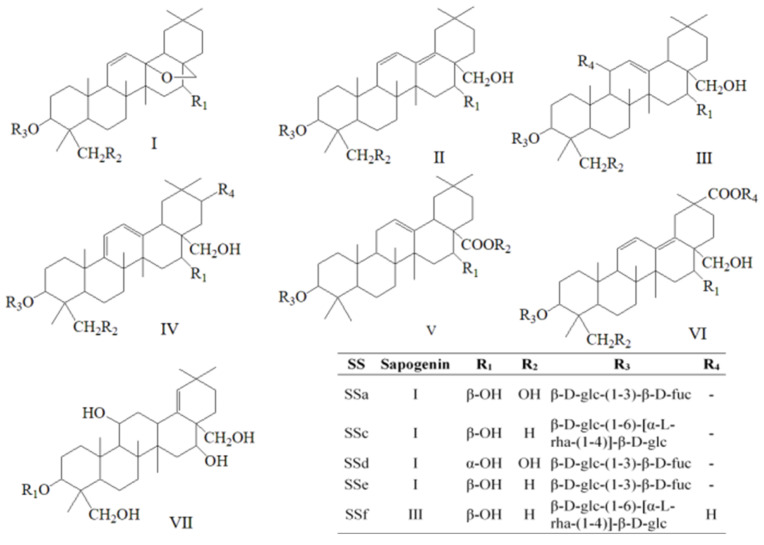
Structure types of saikosaponins.

Radix Bupleuri mainly contains saikosaponins, volatile oils, flavonoids, polysaccharides, fatty acids and sterols [1,6]. Among them, saikosaponins and essential oils are the most prominent active components, the types and contents of which are normally used as the main indexes for quality assessment of Radix Bupleuri [7]. Pharmacological studies have indicated that saikosaponins possess various pharmacological functions, such as immunoregulatory, anti-inflammatory, antioxidant, anti-fibrotic, and anti-hepatoma properties [8,9,10,11]. Saikosaponins mainly belong to pentacyclic triterpenoid derivatives, which can be classified into seven types according to their different aglycones [12]: epoxy ether at C_13_, C_28_-position (type I), isocyclic diene (type II), C_12_-ene (type III), homocyclic diene (type IV), C_12_-ene-C_28_-carboxylic acid (type V), isocyclic diene and C_30_-carboxylic acid (type VI), and C_18_-ene (type VII), the structural formulas of which are shown in Figure 1. More than 100 kinds of saikosaponins have been discovered so far, among which saikosaponin a (SSa) and saikosaponin d (SSd) have attracted much attention for their good activity and high content in Radix Bupleuri. Chinese Pharmacopoeia also prescribes their contents as the measurement index of total saponin content in Radix Bupleuri [13]. However, a growing number of saikosaponins are found to be of high content and good activity as well. Additionally, the precise analysis methods that are applicable to more kinds of saikosaponins are demanded increasingly for further pharmacological research.

On the other hand, much of the current literature on the activity research of Radix Bupleuri pays particular attention to the volatile oils, which mainly refer to monoterpenes and sesquiterpenes [14]. In addition to playing physicochemical roles, they exhibit considerable pharmacological activities, such as clear antipyretic, analgesic, anti-inflammatory and anti-convulsant effects [15], making them suitable to be used for medicinal purposes. Radix Bupleuri injections and transdermal patches, which are formulated with volatile oils, have been widely used in the clinical setting. Although they only account for about 0.15% of the root weight, the volatile oils are an essential active component and play an important role in the therapeutic effects and quality evaluation of Radix Bupleuri.

A comprehensive and precise detection of the complex Radix Bupleuri extracts is the premise on which quality evaluation and difference analysis are based. High-performance liquid chromatography (HPLC) and gas chromatography (GC) coupled with mass spectrometry (MS) methods have proven to be applicable in the qualitative analysis of saponins and volatile oils, as they can rapidly separate many complex compounds and provide unique retention times as well as structural information [16,17]. Another advantage of using the chromatography-based MS method is that it is suited for almost all polar and non-polar organic compounds. As for quantitative analysis, MS in multiple reaction monitoring (MRM) mode also offers an effective way. It avoids the interference of co-eluting components through exact scanning of the predefined precursor and product ions, and therefore, has attractive features of good accuracy, precision, and repeatability in the quantification of bioactive metabolites. We showed previously the simultaneous quantification of 14 ginsenosides in cultivated ginseng using an established HPLC-MRM/MS method, which provided insights into the accumulation characteristics of ginsenoside affected by cultivation region and age [18].

Previous research has been devoted to the analysis of constituents in different varieties of Radix Bupleuri, such as fatty acids, lipids, and saponins [19,20]. Tykheev investigated the chemical composition of the essential oils and lipid fraction of B. scorzonerifolium from different habitats [21]. Saracoglu analyzed the fatty acid composition and the oil content in five endemic *Bupleurum* species, which proved the significant nutritional value of *Bupleurum* oils [22]. However, the quantitative analysis of rare saikosaponins as well as the effect of the cultivation region on the accumulation of saponins and volatile compounds in Radix Bupleuri have rarely been reported, especially for Radix Bupleuri grown in the main production regions of northwest China.

Herein, the effect of cultivation region on the active components of cultivated Radix Bupleuri was systematically investigated. Samples of two-year-old Radix Bupleuri plants were harvested from seven cultivation regions across northwest China. Saikosaponins and volatile compounds were extracted from these samples and were analyzed by HPLC-MS and GC-MS, respectively. Multivariate statistical analysis was further employed to evaluate the differences in these extracted components contributed by the cultivation region based on their respective HPLC-MS and GC-MS profiles, and also to discover the compounds characterized by differential accumulation. Moreover, the quality of Radix Bupleuri samples was assessed by the absolute contents of five saikosaponins, which were accurately quantified by an established HPLC-MRM/MS method. The objective of the current research was to differentiate and evaluate Radix Bupleuri cultivated in different regions and to provide insight into the accumulation characteristics of active components in Radix Bupleuri.

## 2. Results and Discussion

### 2.1. Differential Analysis of Five Saikosaponins in Radix Bupleuri Samples Cultivated in Different Regions

#### 2.1.1. Identification of Five Saikosaponins in Radix Bupleuri 

**Figure 2:**
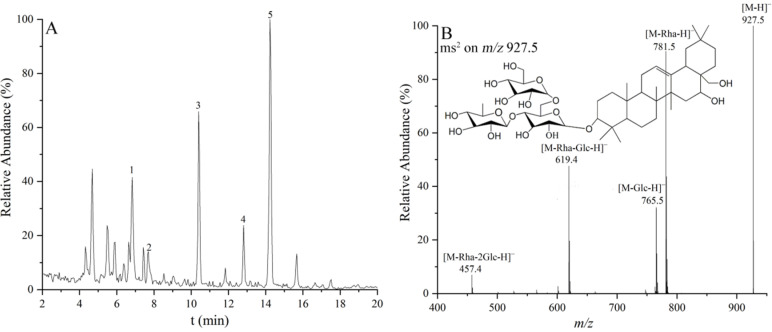
Total ion chromatogram of the saikosaponin extraction of SA Radix Bupleuri sample detected by HPLC-MS in full scan mode (**A**), and MS/MS spectrum of the [M-H]^−^ ion at *m*/*z* 927.5 of peak 2 (**B**).

The negative ion mode in the electrospray ionization (ESI) ion source was used for the qualitative and quantitative analysis of saikosaponins, because it provided more direct structural information and clearer spectra than the positive ion mode [12]. Total ion chromatography (TIC) of the saikosaponins extracted from Radix Bupleuri cultivated in SA is shown as typical in Figure 2A. The peaks of the concerned saikosaponins were labeled 1–5. Their structural identification was first performed by tandem mass spectrometry as well as by comparing their retention behavior with authentic standards. In the negative ion mode, the neutral saponin molecules usually lose a proton to form [M-H]^−^ ion or loosely combine with a formate ion to form [M+HCOO]^−^ ion. This pair of quasi-molecular ions can be used to calculate the relative molecular mass and to infer the molecular formula of saponins. The deprotonation process is endothermic and reduces the internal energy of saponin molecules. It results in the need to obtain additional energy and limited chemical bond cleavage upon collision-induced dissociation. Therefore, the tandem MS analysis of the saponins in the negative ion mode was mainly observed for the product ions resulting from the cleavage of glycosidic bonds [23]. These ions can provide valuable information on the kind of glycosyl substituent.

Taking peak 2 as an example, its relative molecular mass was calculated to be 928.5, and the empirical molecular formula was C_48_H_80_O_17_. Four product ions at *m*/*z* 781.5, *m*/*z* 765.5, *m*/*z* 619.4, and *m*/*z* 457.4 were observed in the MS/MS spectrum of its [M-H]^−^ ion at *m*/*z* 927.5, as shown in Figure 2B. The neutral loss of 162 Da and 146 Da between the four ions indicated the presence of two hexose and one deoxyhexose substituents in the structure of peak 2. Therefore, these ions were assignable to [M-Rha-H]^−^, [M-Glc-H]^−^, [M-Rha-Glc-H]^−^, and [M-Rha-2Glc-H]^−^ ions, respectively. The coexistence of the [M-Rha-H]^−^ and [M-Glc-H]^−^ ions demonstrated the presence of two terminal substituents, namely rhamnose and glucose residues. Moreover, the intensity of the [M-Rha-H]^−^ ion was significantly higher than that of the [M-Glc-H]^−^ ion, suggesting that the rhamnose terminal was more easily dissociated than the glucose terminal. Therefore, as compared to the authentic standard, peak 2 was clearly attributed to saikosaponin f (SSf), and the other four peaks were further identified as saikosaponin c (SSc), SSa, saikosaponin e (SSe), and SSd sequentially, whose structures and spectra information are shown in Figure 1 and Table 1. The above structural analysis rationalized the results of the MRM optimization, indicating that product ions were mainly generated by glycosyl fragmentation, as well as the selection of product ion I and II. Two ion pairs, that is precursor ion combined with product ion I and product ion II, respectively, are usually selected to improve the accuracy of quantification in MRM analysis, where the product ion I is usually the ion of highest intensity and also used for the qualitative analysis of the target compound. For example, the [M-Rha-H]^−^ ion of SSf was selected as the dominant product ion I rather than the [M-Glc-H]^−^ ion, as shown in the Materials and Methods section.

At present, more than 100 saikosaponins have been isolated from Radix Bupleuri and its processed products, but most of their contents are too low in the original medicinal materials to be easily extracted and separated [24]. In this study, SSa, SSc, SSd, SSe, and SSf were chosen for the quantitative experiments. This is because SSa, SSc, and SSd are the representative saikosaponins with high content and good pharmacological activity. On the other hand, SSe and SSf were reported to have good immunomodulatory effects, indicating that they are potential pharmaceutically active candidates for traditional Chinese medicine [25].

Saikosaponins were extracted from Radix Bupleuri using a developed ultrasonic extraction method. The parameters of extraction time, solvent, and solid/liquid ratio that affect the total contents of the five saikosaponins were investigated under different conditions. As shown in Figure S1, the optimized extraction conditions were found at an extraction time of 60 min and solid/liquid ratio of 50 mg/g using 15% ammonia methanol solution. Compared with the traditional heat-reflux extraction method, the established ultrasonic extraction method was more convenient, efficient, and time-saving, and could protect the active components from decomposition [26]. On the other hand, volatile compounds were extracted by the steam distillation method recorded in Chinese Pharmacopoeia without developing and optimizing a new method. This is because among the few extraction methods for volatile components from Radix Bupleuri, steam distillation is recognized as the most effective one. In addition, there have been many reports on the extraction of volatile components using steam distillation [27,28].

#### 2.1.2. Validation of Quantitative Method

As observed in Figure 2A, most of the saikosaponins could be well separated within 20 min, providing a basis for the accuracy of the HPLC-based quantitative method. The linearity, precision, repeatability, stability, and recovery of the established HPLC-MRM/MS method were evaluated to validate the feasibility of quantitative analysis. As shown in Table 2, the calibration curves of the five saikosaponins had good linearity in a wide range of up to three orders of magnitude with correlation coefficients (R^2^) greater than 0.9940. Moreover, the limit of detection (LOD) and limit of quantification (LOQ) were as low as 0.005 and 0.017 μg/mL, respectively, indicating a good sensitivity for the detection of saikosaponins. As Table 3 shows, the intraday and interday precision, repeatability, and stability of the five saikosaponins were all less than 4%. Additionally, the average recoveries were between 95.55% and 103.20% with relative standard deviation (RSD) of less than 4% on the three amount levels. Together, these validated results proved the developed HPLC-MRM/MS method to be suitable for the simultaneous determination of the five saikosaponins in Radix Bupleuri extract.

#### 2.1.3. Quantitative Analysis of Five Saikosaponins in Radix Bupleuri Cultivated in Different Regions

**Figure 3:**
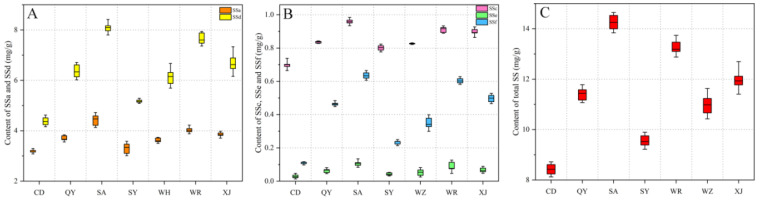
Boxplots of the SSa and SSd (**A**), SSc, SSe, and SSf (**B**), and total saikosaponin (**C**) contents in Radix Bupleuri harvested from different cultivation regions.

The validated HPLC-MRM/MS method was used for the quantitative determination of the five saikosaponins in Radix Bupleuri samples from seven different regions. The contents of the saponins are expressed as mean ± standard deviation in Table S1 and boxplots in Figure 3, respectively. It can be seen from Figure 3 that the contents of the five saikosaponins varied by cultivation region. Among them, Radix Bupleuri harvested from Shouyang County (SA) in Gansu Province had the highest saikosaponin content, followed by harvested samples from Wanrong County (WR) and Xinjiang County (XJ) in Shanxi Province, Qingyang City (QY) in Gansu Province, Wuzhong City (WZ) in Ningxia Province, and Sanyuan County (SY) in Shaanxi Province, while the samples from Chengde City (CD) in Hebei Province had the lowest saikosaponin content. The five saikosaponins showed the same trend as the total saikosaponins in content between different cultivation regions. Analysis of variance (ANOVA) was conducted to investigate the effect of cultivation region on the contents of individual and total saikosaponins. The results in Table S2 show that the contents of SSa, SSc, SSd, SSe, and SSf as well as their total contents were significantly affected by the cultivation region. Although they were all *Bupleurum* plants, the quality of Radix Bupleuri cultivated in different regions was quite different, which may be attributed to a combination of factors, such as the germplasm of the Radix Bupleuri planted and the climate and soil in the growing area. Moreover, the contents of SSa and SSd accounted for an average of 35.0% and 53.7% of the total saikosaponins, respectively, indicating that they accounted for most of the saponins in Radix Bupleuri. While the sum of the contents of SSc, SSe, and SSf accounted for about 11.3% on average, these three saikosaponins also contributed significantly to the total saponin content and could be used as indicators to evaluate the quality of Radix Bupleuri.

### 2.2. Differential Analysis of Radix Bupleuri from Different Cultivation Regions Based on HPLC-MS and GC-MS Analysis

#### 2.2.1. Principal Component Analysis

**Figure 4:**
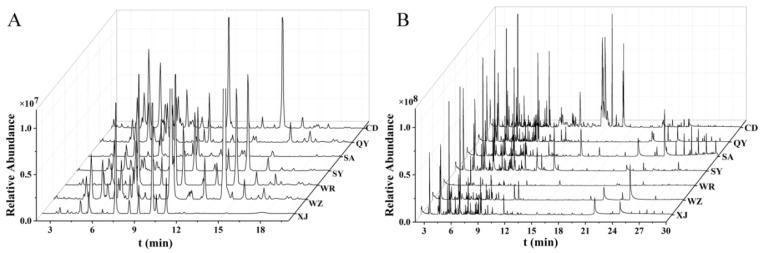
HPLC-MS analysis of the saikosaponins (**A**) and GC-MS analysis of the volatile compounds (**B**) in the Radix Bupleuri samples from different cultivation regions.

The saikosaponins and volatile compounds in the Radix Bupleuri samples from all seven cultivation regions were analyzed by HPLC-MS and GC-MS, respectively, and the resulting TICs are shown in Figure 4. All of these extracts exhibited diverse compositions and complex spectra. In addition, the high similarity of the spectra of saikosaponins made them difficult to be distinguished directly by TIC. Moreover, although the spectra of the volatile compounds were less similar, the differential compounds could not be accurately identified. Therefore, the HPLC-MS and GC-MS datasets were converted and subjected to multivariate statistical analysis to distinguish Radix Bupleuri samples from different cultivation regions and to identify the differential characteristics of saikosaponins and volatile compounds.

**Figure 5:**
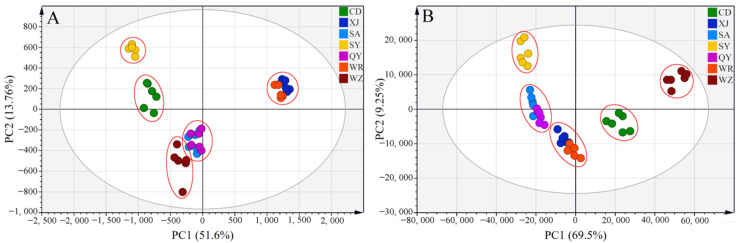
PCA score plots of Radix Bupleuri cultivated in different regions derived from the HPLC-MS datasets of saikosaponins (**A**) and the GC-MS datasets of volatile compounds (**B**).

Principal component analysis (PCA) is an unsupervised pattern recognition technique that preserves the original data as much as possible. It employs a few comprehensive indicators to explain multiple variables with specific correlations in order to reduce the dimensionality of complex data and to observe the correlation between samples. Figure 5A,B illustrate PCA score plots of saikosaponins and volatile compounds extracted from the Radix Bupleuri samples cultivated in different regions, respectively. All of the samples were free of outliers at 95% confidence interval. In Figure 5A, the first and the second principal components described 51.6% and 13.76% of the total variables of the HPLC-MS datasets, respectively, that is, a total of 65.36% of the original variables, while in Figure 5B, they accounted for 78.75% of the total variables of the GC-MS datasets. The Radix Bupleuri samples from the same regions clustered together in Figure 5A,B, indicating the homogeneous quality of these samples that were grown in similar environments. It is worth noting that neither the XJ and WR samples harvested from Shanxi Province nor the SA and QY samples harvested from Gansu Province could be distinguished from each other. This indicated that Radix Bupleuri samples grown in the same province were similar in terms of saikosaponins and volatile compounds. Therefore, all of the samples were clearly divided into five groups according to the province where they were grown, suggesting the Radix Bupleuri samples cultivated in different provinces were significantly different in terms of their saikosaponin and volatile compound contents. In Figure 5A, the WZ samples grown in Ningxia Province are distributed near the SA and QY samples that were cultivated in Gansu Province. This may be attributed to the geographical proximity of Ningxia and Gansu Province, which is conducive to the development of a similar climate and natural environment in these regions. However, the samples from these two regions were separated distinctly in Figure 5B, indicating that cultivation region is only one of many factors that help to determine the chemical composition of Radix Bupleuri.

#### 2.2.2. Hierarchical Cluster Analysis

**Figure 6:**
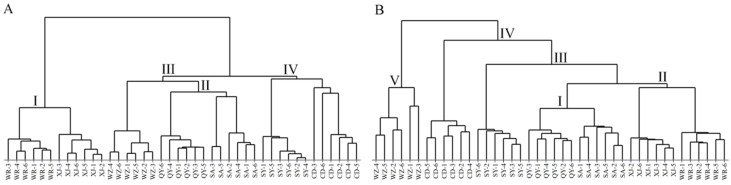
Dendrograms of Radix Bupleuri samples cultivated in different regions constructed by HCA with the HPLC-MS datasets of saikosaponins (**A**) and the GC-MS datasets of volatile compounds (**B**).

Hierarchical cluster analysis (HCA) is another unsupervised pattern recognition method that classifies samples into relatively uniform categories based on the similarity of data [29]. The dendrograms of HCA were generated using the converted HPLC-MS and GC-MS datasets of all of the collected Radix Bupleuri samples, the results of which are shown in Figure 6. All of the samples were able to be classified individually based on their cultivation regions, further demonstrating the high similarity of saikosaponins and volatile compounds in Radix Bupleuri samples cultivated in the same region. In Figure 6A, the WR and XJ samples and the QY and SA samples were first classified as cluster I and II, respectively, indicating that the similarity of saponins in Radix Bupleuri from the same province was higher than that in samples from different provinces. The WZ samples were similar to cluster II, with which they were classified as cluster III. The CD and SY samples were classified in cluster IV and were far away from cluster I. Notably, both WR and XJ were geographically located between SY and CD, but the similarity of the saikosaponins in their samples was not high, indicating that geographical distance was not a decisive factor affecting the similarity of saikosaponins in Radix Bupleuri samples from different cultivation regions. In Figure 6B, all of the samples could still be preferentially classified according to the province where they were grown, showing a high similarity in volatile compounds of Radix Bupleuri from the same province. Cluster I and II gathered from the samples of Gansu and Shanxi Province, respectively, were classified with the SY samples as cluster III and subsequently with the CD samples as cluster IV. Additionally, the WZ samples were included in cluster V alone, indicating that their volatile compounds were less similar to those from the other six regions.

#### 2.2.3. Partial Least Squares-Discriminate Analysis

**Figure 7:**
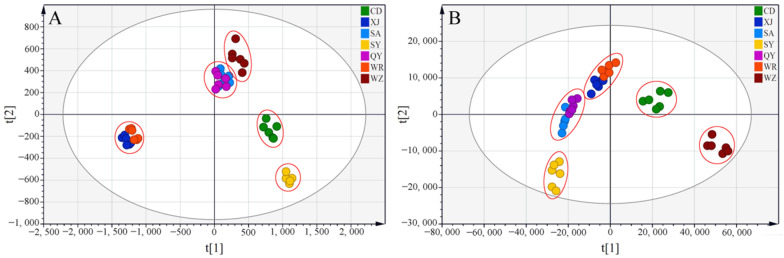
PLS-DA scores plots derived from the HPLC-MS datasets of saikosaponins (**A**) and the GC-MS datasets of volatile compounds (**B**) in Radix Bupleuri cultivated in different regions.

Partial least squares-discriminate analysis (PLS-DA), a supervised pattern recognition technique, was conducted in an effort to discover characteristic differences and composition variations between Radix Bupleuri samples from different regions. Two PLS-DA models were generated first based on the converted HPLC-MS and GC-MS datasets to find the most discriminating features in saikosaponins and volatile compounds of the collected Radix Bupleuri samples, respectively. The quality of the two models was described by R^2^ and Q^2^ values via cross-validation. R^2^ indicates the explanatory power of the model, while Q^2^ indicates the extent to which the model predicts new data. The score plots illustrated the clustering of the Radix Bupleuri samples from different origins with the model parameters of R^2^ = 0.985 and Q^2^ = 0.906 for the HPLC-MS dataset (Figure 7A) and R^2^ = 0.963 and Q^2^ = 0.927 for the GC-MS dataset (Figure 7B), respectively. These parameters revealed high goodness of fit and high predictive ability of both PLS-DA models. Permutation tests were further conducted with 200 random permutations to validate each PLS-DA model. As shown in Figure S1, all of the permuted R^2^ and Q^2^ values were lower than those in the original models, and the regression line of the permuted Q^2^ values intersected with the vertical axis below 0, which indicated the validity of these two models. The XJ and WR samples as well as the SA and QY samples were found to be still indistinguishable in Figure 7 even under the mandatory grouping conditions of PLS-DA, indicating the quality of Radix Bupleuri grown in these two provinces was extremely similar. The samples were distinctly divided into five groups according to the province where they were grown, indicating that the provinces where the Radix Bupleuri samples were grown could be discriminated using these models.

**Figure 8:**
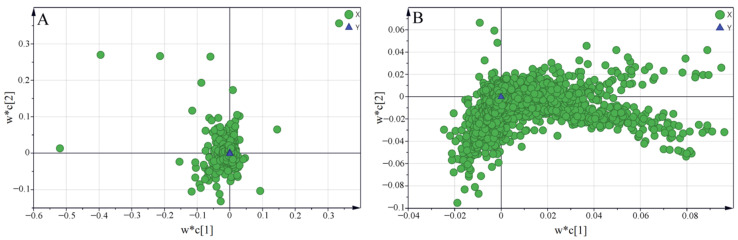
Loading plots of PLS-DA models derived from the HPLC-MS datasets of saikosaponins (**A**) and the GC-MS datasets of volatile compounds (**B**) in Radix Bupleuri.

In order to select the saponins and volatile compounds that contributed significantly to the grouping, variable importance for projection (VIP) values were calculated from the PLS-DA models. VIP values indicate the importance of the variable to explain the dataset and to the critical influence on grouping. In the loading plots, the further away from the origin, the greater the contribution of the variables to the discrimination of groups. By combining the loading plots in Figure 8, saponins and volatile compounds with VIP > 1, were selected as differential components. Finally, fifty-eight volatile compounds were obtained and identified by means of retrieval of MS spectra from the NIST database, while twenty-eight saponins were identified using literature data with references, and the identification of the five quantified saikosaponins was further confirmed using standard comparison. Then, the Student’s *t*-tests showed that these compounds were significantly different (*p* < 0.05) at least between two cultivation regions. The information on the identified compounds is listed in Table 4 and Table 5. Among the twenty-eight saponins, twenty-two belonged to triterpenoid saponins and their derivatives. Additionally, most of the fifty-eight volatile components were aliphatic compounds, as well as some alcohols, aldehydes, and terpenoids. The number of differential components for volatile compounds was much greater than that for saponins, indicating that the cultivation region has a strong influence on the kind and content of volatile components in Radix Bupleuri.

## 3. Materials and Methods

### 3.1. Materials and Chemicals

Forty-two batches of Radix Bupleuri samples that were cultivated for two years were collected during September and October in 2021 from different cultivation regions in China, including Sanyuan County (SY) in Shaanxi Province, Shouyang County (SA) and Qingyang City (QY) in Gansu Province, Xinjiang County (XJ) and Wanrong County (WR) in Shanxi Province, Chengde City (CD) in Hebei Province, and Wuzhong City (WZ) in Ningxia Province. Their botanical origins were all authenticated to *Bupleurum chinense* DC by Professor Shumin Wang of Changchun University of Chinese Medicine, China. Authentic standards of SSa, SSd, SSc, SSe, and SSf were purchased from Shanghai Yuanye Biological Technology Co., Ltd. (Shanghai, China). HPLC-grade methanol, acetonitrile, and n-hexane were acquired from Tedia Company, Inc. (Fairfield, CT, USA), while formic acid was purchased from Thermo Fisher Scientific (Waltham, MA, USA). Analytical grade methanol, ammonia, and n-butanol were bought from Beijing Chemical Industry Group Co, Ltd. (Beijing, China). Ultrapure water was prepared by Milli-Q water purification apparatus (Merck Millipore, Bedford, OH, USA).

### 3.2. Preparation of Sample and Standard Solutions

#### 3.2.1. Extraction of Saikosaponins from Radix Bupleuri

All of the Radix Bupleuri samples were washed, dried in the shade, and then pulverized to 40 mesh. A 0.5 g amount of Radix Bupleuri powder was accurately weighed and mixed with 25 mL of 15% ammonia-methanol solution. The solution was extracted using an ultrasonic bath for 60 min at room temperature followed by filtration. The extraction procedure was repeated twice in the same conditions. The filtrate from two extractions were combined and dried through rotary evaporation. The resulting residue was dissolved in 5 mL of n-butanol. After centrifugation at 7000 rpm for 5 min, the supernatant was evaporated to dryness and then fully redissolved in HPLC-grade methanol to a constant volume.

#### 3.2.2. Extraction of Volatile Compounds from Radix Bupleuri

Volatile compounds were extracted from Radix Bupleuri by steam distillation. Typically, 25.0 g of Radix Bupleuri powder was immersed in 250 mL of ultrapure water for 12 h. The volatile oil determination apparatus was filled with water and sealed with 2 mL of n-hexane. After steam distillation for 8 h, the n-hexane layer was collected and dehydrated using anhydrous magnesium sulfate. The solution was then transferred to a 10 mL volumetric flask and filled up with n-hexane.

#### 3.2.3. Preparation of Standard Solutions

The single standard solutions were prepared by individually dissolving accurately weighed 1.0 mg of authentic saikosaponin standard in 10 mL of HPLC-grade methanol, and then were used to optimize the MRM parameters, including the precursor and product ions, as well as the collision energy. All of the saikosaponin standards were dissolved in 2 mL of methanol to prepare the mixed standard stock solutions, which were diluted into a series of mixed standard solutions at different concentration levels to establish the calibration curves. All of the prepared samples and standard solutions were filtered through a 0.22 μm filter membrane prior to MS analysis.

### 3.3. Chromatography-Based MS Conditions

#### 3.3.1. HPLC-MS Conditions

HPLC analysis was performed on an Ultimate 3000 system (Thermo Fisher Scientific Inc, San Jose, CA, USA) equipped with a quaternary bump, an online vacuum degasser, an autosampler, and a thermostatically controlled column compartment that worked at the temperature of 25 °C. A Thermo Syncronis C_18_ column (100 mm × 2.1 mm, 1.7 μm) was used to separate the analytes with the following gradient elution: 0–20 min, 35–90% A; 20–20.5 min, 90–35% A; 20.5–25 min, 35% A, where the mobile phase A and B were acetonitrile and 0.1% (*v*/*v*) formic acid aqueous solution, respectively. The analytes were delivered at a flow rate of 0.2 mL/min. The injected sample volume was 2 μL.

MS analysis was performed using a TSQ Endura triple quadrupole mass spectrometer (Thermo Fisher Scientific Inc., San Jose, CA, USA), which was coupled with HPLC apparatus via an ESI ion source. Nitrogen was supplied as sheath gas, auxiliary gas, and sweep gas in the ion source at a flow rate of 35, 10, and 1 arbitrary units. The analytes were detected in the negative ion mode with optimized capillary temperature, spray voltage, and vaporizer temperature at 325 °C, −2500 V, and 290 °C. Ultrapure argon was used as the collision gas for the collision-induced dissociation experiment. The optimization of MRM parameters was performed using the built-in program when introducing a single standard solution into the ion source via an infusion pump. Two ion pairs and the corresponding collision energy were selected for quantification (Table 6). Full scan mode was run in the range of *m*/*z* 100 to 2000 with a scan speed of 1000 Da/s, and data were collected automatically in centroid mode.

#### 3.3.2. GC-MS Conditions

GC-MS analysis was performed using a Trace 1310 gas chromatograph coupled with TSQ 8000 triple quadrupole mass spectrometer (Thermo Fisher Scientific Inc., San Jose, CA, USA) equipped with an HP-5MS capillary column (30 m × 0.25 mm, 0.25 μm) (Agilent Technologies, Santa Clara, CA, USA). Ultrapure helium was used as the carrier gas with a flow rate of 1.2 mL/min. The temperatures of injector, MS transfer line, and ion source were set as 250 °C, 230 °C, and 230 °C, respectively. The injection volume was 1 μL, which was split in the ratio of 20:1. The oven temperature was programmed to hold initially at 60 °C for 2 min, then increase from 60 °C to 140 °C at 15 °C/min followed by a hold at 140 °C for 2 min, then increase from 140 °C to 180 °C at 5 °C/min followed by a hold at 180 °C for 3 min, and then increase from 180 °C to 250 °C at 10 °C/min with a final hold at 250 °C for 10 min. MS was performed in full scan mode with the mass range of *m*/*z* 100 to 700, using an electron impact ion source operated at 70 eV.

### 3.4. Validation of Methodology

The calibration curves of saikosaponins were established by linearity fitting of eight mixed standard solutions of different concentrations. The LOD and LOQ were determined at the signal to noise ratios of 3 and 10, respectively. The stability was validated by analyzing the variations in saikosaponin content in a freshly extracted sample solution from 0 h to 24 h at 4 h intervals. Variations were expressed by RSD. The precision was evaluated by the intraday and interday variations of the saikosaponin content in extracted samples, which were measured six times in one day and were measured three times a day for three consecutive days. One Radix Bupleuri sample was divided into six equal parts, with the saikosaponin in each extracted and analyzed to evaluate the repeatability based on the variations in saikosaponin content between each part. The accuracy was assessed by the recovery of the spiked saikosaponin standards from Radix Bupleuri powder. The standards were spiked at three amount levels, which were approximately equal to 80%, 100%, and 120% of the original content of each saikosaponin in the extract, and three replicates of each amount level were tested. The recovery was calculated as follows: recovery (%) = 100 × (found content-original content)/spiked content.

### 3.5. Data Processing and Statistical Analysis

The peak areas in TIC of the detected saikosaponins were integrated automatically using Qual Browser in Xcalibur software (version 2.2 SP1.48, Thermo Fisher Scientific Inc, San Jose, CA, USA). The saikosaponin contents were calculated by substituting the peak areas into the established calibration curve equations. Each Radix Bupleuri sample was quantitatively analyzed in triplicate. The saikosaponin contents were expressed as mean and standard deviation and subjected to analysis of variance (ANOVA) and box-plot analysis using OriginPro software (version 9.1, OriginLab Corporation, Northampton, MA, USA). HPLC-MS and GC-MS data were recorded automatically by Xcalibur software and processed by SIEVE software (Version 2.1, Thermo Fisher Scientific Inc., San Jose, CA, USA) for peak matching, peak alignment, and peak area normalization. Additionally, a data table containing sample name, retention time, *m*/*z*, and peak intensity was obtained. Data from isotope peaks and missing values greater than 80% were removed. The remaining data were imported into SIMCA-P software (Version 14.1, Umetrics, Umeå, Sweden) and converted for multivariate statistical analysis, including PCA, HCA, and PLS-DA.

### 3.6. General Process of Compound Identification

The saponins were analyzed by HPLC-MS in negative ion mode of ESI ion source to obtain the information on the retention time and the quasi-molecular ion pair of [M-H]^−^ ion and [M+HCOO]^−^ ion, which were used to calculate the relative molecular mass and infer the molecular formula. Then, tandem MS analysis was performed using collision-induced dissociation at different collision energies to observe the fragment ions. Based on the neutral loss between fragment ions, the number and type of glycosyl substituents in saponins were summarized. The characteristic aglycone ion observed at high collision energy was indicative of the types of saponins. Combining the above structural information with the literature data, the structure of saponins could be identified. For the identification of volatile compounds, the obtained GC-MS data were directly subjected to NIST 14 database to search for the matching compounds by comparing their mass spectra.

## 4. Conclusions

The present study was designed to determine the effect of cultivation region on the accumulation of saponins and volatile compounds in Radix Bupleuri. As a proof of concept, forty-two batches of Radix Bupleuri samples were harvested from seven regions in northwest China and processed to extract saikosaponins and volatile compounds, which were analyzed by HPLC-MS and GC-MS, respectively. The results of quantitative analysis showed that the cultivation region made a significant difference on the content of SSa, SSc, SSd, SSe, and SSf, which were determined through an established HPLC-MRM/MS method. The Radix Bupleuri grown in SA county had the highest content of saikosaponins among all of the collected samples. In addition, the HPLC-MS and GC-MS datasets contained variables that related to the cultivation regions. The Radix Bupleuri samples were able to be discriminated based on their cultivation regions using multivariate statistical analysis of the chromatography-based MS datasets. PCA and HCA confirmed significant differences between samples of Radix Bupleuri cultivated in different provinces, whereas samples from the same province showed a significant similarity to each other. Twenty-eight saponins and fifty-eight volatile compounds were identified as the differentially accumulated components that contributed significantly to the discrimination of cultivation regions. These results suggest the approach in the present study will prove useful in discriminating the cultivation regions of Radix Bupleuri. Moreover, the cultivation region is one of the critical factors affecting the quality of Radix Bupleuri, and volatile compounds are also important indexes for its quality assessment. Considerably more work will need to be done to elucidate the effect of regional difference on the active components in Radix Bupleuri from the perspective of plant growth and metabolic pathways combined with climate and geographical factors.

## Figures and Tables

**Table 1:** Chemical information and major product ions of the five saikosaponins.

Peak	t_R_ (min)	Identification	Measured Mass	Molecular Formula	Product Ions
1	6.83	SSc	926.5	C_48_H_78_O_17_	925.5 [M-H]^−^, 779.5 [M-Rha-H]^−^, 763.5 [M-Glc-H]^−^, 617.4 [M-Rha-Glc-H]^−^, 455.6 [M-Rha-2Glc-H]^−^
2	7.69	SSf	928.5	C_48_H_80_O_17_	927.5 [M-H]^−^, 781.5 [M-Fuc-H]^−^, 619.4 [M-Fuc-Glc-H]^−^, 457.4 [M-Fuc-2Glc-H]^−^
3	10.39	SSa	780.5	C_42_H_68_O_13_	779.5 [M-H]^−^, 617.4 [M-Glc-H]^−^, 471.4 [M-Glc-Fuc-H]^−^
4	12.81	SSe	764.5	C_42_H_68_O_12_	763.5 [M-H]^−^, 601.4 [M-Glc-H]^−^, 455.4 [M-Rha-2Glc-H]^−^
5	14.22	SSd	780.5	C_42_H_68_O_13_	779.5 [M-H]^−^, 617.4 [M-Glc-H]^−^, 471.6 [M-Glc-Fuc-H]^−^

**Table 2:** Calibration curve, R^2^, and linear range of the five saikosaponins detected by the HPLC-MRM/MS method.

Saikosaponin	Calibration Curve	R^2^	Linear Range (μg/mL)	LOD (μg/mL)	LOQ (μg/mL)
SSa	y = 13,888.3 + 92,655.3x	0.9986	0.1–100.0	0.009	0.030
SSc	y = 64,383 + 755,798x	0.9974	0.05–10.0	0.006	0.020
SSd	y = 36,478.4 + 89,231.8x	0.9953	0.1–100.0	0.015	0.050
SSe	y = 133,789 + 313,062x	0.9949	0.03–3.0	0.009	0.030
SSf	y = 46,408.3 + 909,911x	0.9966	0.05–10.0	0.005	0.017

**Table 3:** Precision, repeatability, stability, and recovery of the five saikosaponins detected by the HPLC-MRM/MS method.

Saikosaponin	Intraday Precision RSD (*n* = 9, %)	Interday Precision RSD (*n* = 6, %)	Repeatability RSD (*n* = 6, %)	Stability RSD (*n* = 7, %)	Recovery (%)/RSD (*n* = 3, %)		
					80%	100%	120%
SSa	2.93	1.78	2.04	1.98	101.32/1.57	101.96/2.03	99.63/2.19
SSc	2.06	1.90	1.68	1.53	100.91/1.95	102.46/1.47	95.55/2.09
SSd	1.53	2.23	1.46	1.46	101.13/1.32	102.45/1.22	98.10/1.82
SSe	3.14	2.77	3.23	3.28	97.47/1.65	103.20/2.59	98.46/1.48
SSf	3.45	3.23	3.18	2.89	98.83/2.61	102.05/1.78	96.12/2.57

**Table 4:** Information on the differential saponins from Radix Bupleuri cultivated in different regions analyzed by HPLC-MS.

No.	t_R_ (min)	VIP	Compound Name	[M-H]^−^ Ion (*m*/*z*)	Fragment Ions (*m*/*z*)	Molecular Formula	References
1	4.01	2.0	3′,4′-dimethoxy quercetin	329	314, 299	C_18_H_34_O_5_	[30]
2	4.17	1.3	hydroxy-SSc or its isomer	943	811, 649, 503, 471, 453	C_48_H_80_O_18_	[12]
3	4.49	1.6	rotundioside w	941	795, 777	C_48_H_78_O_18_	[31]
4	4.86	1.6	hydroxy-SSa	797	635, 559, 489	C_42_H_70_O_14_	[12]
5	6.57	3.3	SSi	925	779, 763, 617	C_48_H_78_O_17_	[12]
6	6.69	3.3	SSc	971	925, 779, 763, 617, 455	C_48_H_78_O_17_	[12]
7	6.77	1.2	rotundioside n	941	779, 763, 618	C_48_H_78_O_18_	[31]
8	7.14	1.7	hydroxy-SSd	797	635, 559, 489	C_42_H_70_O_14_	[12]
9	7.56	5.2	SSf	973	927, 781, 765, 619, 457	C_48_H_80_O_17_	[12]
10	8.72	1.3	rotundioside p	943	811, 649, 503, 471	C_48_H_80_O_18_	[12]
11	9.36	4.2	2″-O-acetyl SSb3	853	811, 793, 649	C_45_H_74_O_15_	[32]
12	10.33	2.5	SSa	825	779, 617, 541, 471	C_42_H_68_O_13_	[12]
13	10.5	13.4	SSb2	825	779, 617, 541, 471	C_42_H_68_O_13_	[12]
14	11.11	5.2	isomer of SSf	927	781, 765, 619	C_48_H_80_O_17_	[12]
15	11.28	1.6	chinoposaponin XVIII	941	779, 617	C_48_H_78_O_18_	[7]
16	11.43	1.2	2″-O-acetyl SSa	821	779, 761, 617	C_44_H_70_O_14_	[12]
17	11.49	1.0	diacetyl SSa	863	821, 761	C_46_H_72_O_15_	[30]
18	11.54	1.5	3″-O-acetyl SSa	821	779, 761, 617, 541, 471	C_44_H_70_O_14_	[30]
19	11.66	1.5	malonyl-SSa	865	821, 779, 761, 617	C_45_H_70_O_16_	[7]
20	11.85	12.0	SSb1	825	779, 617, 471	C_42_H_68_O_13_	[12]
21	11.89	2.3	3β, 23, 28-trihydroxyolean-11, 13(18)-diene-16-one 3-O-β-D-glucopyranosyl-(1-3)-β-D-fucopyranoside	777	615, 539, 469, 437	C_42_H_66_O_13_	[12]
22	11.92	3.6	acetyl SSa	821	779, 617	C_44_H_70_O_14_	[31]
23	12.75	3.9	SSe	809	763, 601, 455	C_42_H_68_O_12_	[12]
24	13.84	1.2	3″-O-acetyl SSb2	821	779, 761, 617	C_44_H_70_O_14_	[12]
25	14.14	2.4	SSd	825	779, 617	C_42_H_68_O_13_	[12]
26	15.51	1.5	malonyl-SSd	865	821, 779, 761, 617	C_45_H_70_O_16_	[32]
27	16.07	1.0	diacetyl SSd	863	821, 779, 76	C_46_H_72_O_15_	[30]
28	16.13	1.5	6″-O-acetyl SSd	821	779, 761, 617	C_44_H_70_O_14_	[12]

**Table 5:** Information on the differential volatile compounds from Radix Bupleuri cultivated in different regions analyzed by GC-MS.

No.	t_R_ (min)	VIP	Compound Name	M^+^• Ion (*m*/*z*)	Molecular Formula
1	2.02	1.4	L-isoleucine	130.1	C_6_H_13_NO_2_
2	2.02	1.4	3, 4-dimethyl-1-hexene	111.2	C_8_H_16_
3	2.55	1.2	cyclobutene, 2-propenylidene	91.1	C_7_H_8_
4	2.56	1.0	n-hexane	85.2	C_6_H_14_C_6_H_14_
5	2.85	1.6	2, 2-dimethyl heptane	127.3	C_9_H_20_
6	2.88	1.6	2-(1-methylbutyl)-oxirane	113.2	C_7_H_14_O
7	4.03	1.3	chloromethyl 2-chloroundecanoate	268.2	C_12_H_22_C_l2_O_2_
8	4.03	1.3	2, 4, 6-trimethyl-heptane	141.3	C_10_H_22_
9	4.07	1.1	2, 5, 6-trimethyl-decane	183.4	C_13_H_28_
10	4.07	1.1	trimethylene oxide	57.1	C_3_H_6_O
11	4.40	1.1	hexyl-oxirane	71.1	C_4_H_8_O
12	4.40	1.1	D-sphingosine	298.5	C_18_H_37_NO_2_
13	4.40	1.1	1-methyl-4-(1-methylethenyl)- 2-cyclohexene	135.2	C_10_H_16_
14	4.8	1.2	phenacyl thiocyanate	176.2	C_9_H_7_NOS
15	4.95	1.6	2-butenoic acid, 3-methylbutyl ester	155.2	C_9_H_16_O_2_
16	4.95	1.5	5-methyl-2-hexanamine	114.2	C_7_H_17_N
17	5.02	2.0	α-pinene	135.2	C_10_H_16_
18	5.05	1.7	4-octyl acetate	171.3	C_10_H_20_O_2_
19	5.05	1.7	4, 4-dimethyl-1-hexene	111.2	C_8_H_16_
20	5.05	1.7	ethyl-cyclohexane	111.2	C_8_H_16_
21	5.09	1.4	di-*t*-butylacetylene	137.3	C_10_H_18_
22	5.24	1.4	4-methyl-cyclopentadecanone	237.4	C_16_H_30_O
23	5.36	1.3	trans-4, 5-epoxydecane	155.3	C_10_H_20_O
24	5.81	1.7	7-methylene-tridecane	195.4	C_14_H_28_
25	5.81	1.7	benzyl 2-chloroethyl sulfone	217.7	C_9_H_11_ClO_2_S
26	5.87	1.7	n-nonane	267.5	C_19_H_40_
27	6.10	1.0	acetophenone	119.2	C_8_H_8_O
28	6.16	2.0	1-nitro-2-octanone	172.2	C_8_H_15_NO_3_
29	6.17	2.1	1-heptadecyne	235.4	C_17_H_32_
30	6.19	1.1	cis-linaloloxide	169.3	C_10_H_18_O_2_
31	6.31	1.6	heptanoic acid	128.2	C_7_H_13_O_2_
32	6.35	1.6	cyclopropylacetic acid	99.1	C_5_H_8_O_2_
33	6.86	1.2	1-(3, 7-dimethyl-1-octenyl)-cyclopropanol	195.3	C_13_H_24_O
34	6.90	1.4	benzyl nitrile	116.2	C_8_H_7_N
35	7.31	1.1	1-(ethenylthio)-octane	171.3	C_10_H_20_S
36	7.32	1.0	1, 2, 3, 4, 5-cyclopentanepentol	149.1	C_5_H_10_O_5_
37	7.61	1.1	1, 4-dimethyl-adamantane	163.3	C_12_H_20_
38	7.90	1.4	1-tridecanol	199.4	C_13_H_28_O
39	8.03	1.1	1-pentanol	87.2	C_5_H_12_O
40	8.11	1.3	methyl-cycloheptane	111.2	C_8_H_16_
41	8.11	1.3	1, 2-dimethyl-cyclohexane	111.2	C_8_H_16_
42	8.36	1.1	2, 4-bis(diazo)adamantane	187.2	C_10_H_12_N_4_
43	8.36	1.1	isopulegol	153.3	C_10_H_18_O
44	8.6	1.9	cyclopentadecanol	225.4	C_15_H_30_O
45	8.68	1.3	2, 5-diethylphenol	149.2	C_10_H_14_O
46	8.69	1.1	thymol	149.2	C_10_H_14_O
47	8.70	1.0	3-methyl-5-(1-methylethyl)-phenol methylcarbamate	206.3	C_12_H_17_NO_2_
48	9.11	1.1	2-carbonitrile-cyclopentanone	108.1	C_6_H_7_NO
49	9.18	1.5	2-undecenal	167.3	C_11_H_20_O
50	9.68	1.3	n-decanoic acid	171.3	C_10_H_20_O_2_
51	9.68	1.3	cubenol	221.4	C_15_H_26_O
52	9.70	1.1	3-methyl-heptanedioic acid dimethyl ester	201.2	C_10_H_18_O_4_
53	9.73	1.3	chamigrene	203.4	C_15_H_24_
54	9.80	1.0	cyclodecane	139.3	C_10_H_20_
55	9.94	1.4	15(S)-hydroxy-(5Z, 8Z, 11Z, 13E)- eicosatetraenoic acid	319.5	C_20_H_32_O_3_
56	9.95	1.0	1, 8-cyclopentadecadiyne	201.3	C_15_H_22_
57	23.2	2.9	12, 15-octadecadienoic acid, methyl ester	293.5	C_19_H_34_O_2_
58	24.06	3.1	linoleic acid ethyl ester	307.5	C_20_H_36_O_2_

**Table 6:** The ion pair list of the precursor ions, product ions and collision energy of the five saikosaponins.

Saikosaponin	Precursor Ion (*m*/*z*)	Product Ion I (*m*/*z*)/Collision Energy (eV)	Product Ion II (*m*/*z*)/Collision Energy (eV)
SSa	779.5	439.6/52.0	617.3/32.2
SSc	971.5	779.3/38.9	925.4/23.7
SSd	779.5	439.2/55.0	617.3/33.1
SSe	809.5	601.3/33.0	763.3/21.3
SSf	973.5	781.3/39.9	927.4/23.4

## Data Availability

Not applicable.

## Supplementary Materials

The following supporting information can be downloaded at: https://www.mdpi.com/article/10.3390/molecules27154830/s1, Figure S1: Optimization of the parameters of extraction time (A), concentration of solvent (B), and solid-liquid ratio (C) for the developed ultrasonic extraction method investigated in different conditions; Figure S2: Permutation tests of PLS-DA models derived from the HPLC-MS datasets of saikosaponins (A) and the GC-MS datasets of volatile compounds (B) in the Radix Bupleuri samples; Table S1: The contents of the five and total saikosaponins in Radix Bupleuri that were cultivated in different regions; Table S2: ANOVA of the effects of cultivation region on saikosaponin content.
