# Supplementary material for: Quantitative Analysis and Differential Evaluation of Radix Bupleuri Cultivated in Different Regions Based on HPLC-MS and GC-MS Combined with Multivariate Statistical Analysis
Source: Molecules. 2022 Jul 28;27(15):4830. doi: 10.3390/molecules27154830 (PMC9369679; doi:10.3390/molecules27154830)
Supplement: Supplementary file 1 [file molecules-27-04830-s001.zip › molecules-1814923-supplementary.pdf]

Supplementary materials

Quantitative analysis and differential evaluation of Radix Bupleuri cultivated in different regions based on HPLC-MS and GC-MS combined with multivariate statistical analysis

Zhenhuan Wang #, Huanxi Zhao #, Lu Tian, Mengya Zhao, Yusheng Xiao, Shuying Liu *, Yang Xiu *

Jilin Ginseng Academy, Changchun University of Chinese Medicine, Changchun 130117, China; 847637379@qq.com (Z.W.); phoenix8713@sina.com (H.Z.); tianlulu9903@163.com (L.T.); 763846780@qq.com (M.Z.); 1660889276@qq.com (Y.X.)

# Zhenhuan Wang and Huanxi Zhao contributed equally to this paper.

* Correspondence: ys830805@sina.com; Tel.: +86-431-86763985 (Y.X); syliu@ciac.ac.cn; Tel.: +86-431-86763978 (S.L)

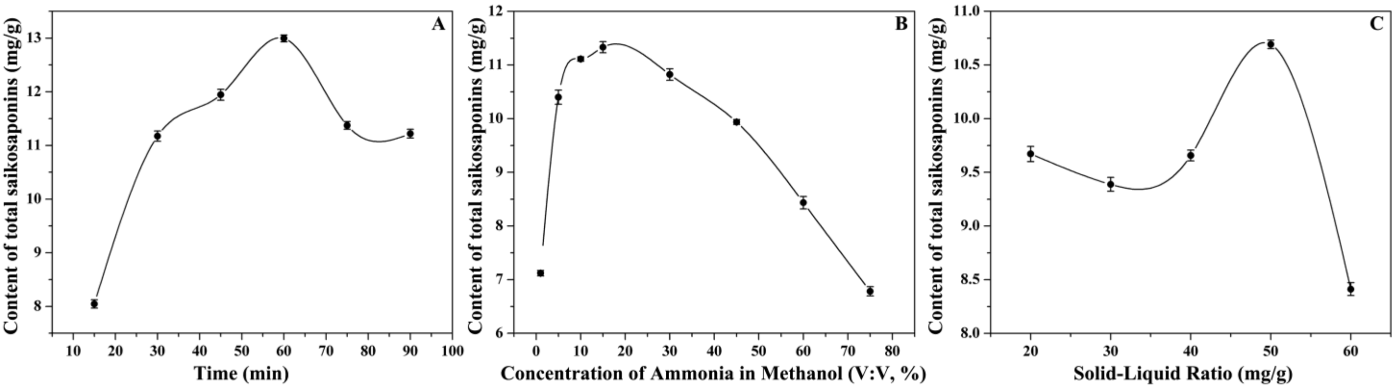

Figure S1. Optimization of the parameters of extraction time (A), concentration of solvent (B), and solid-liquid ratio (C) for the developed ultrasonic extraction method investigated in different conditions

Table S1. The contents of the five and total saikosaponins in Radix Bupleuri that were cultivated in different regions

No.	Content of saikosaponin (μg/mg)					
	SSa	SSc	SSd	SSe	SSf	Total
SY1	3.113±0.007	0.793±0.009	5.236±0.022	0.047±0.004	0.249±0.009	9.427±0.050
SY2	3.299±0.003	0.807±0.007	5.158±0.040	0.035±0.004	0.243±0.009	9.542±0.046
SY3	3.058±0.022	0.798±0.011	5.045±0.047	0.046±0.006	0.234±0.007	9.247±0.051
SY4	3.242±0.036	0.788±0.009	5.028±0.068	0.036±0.004	0.229±0.013	9.322±0.061
SY5	3.483±0.017	0.796±0.012	5.093±0.048	0.041±0.002	0.243±0.007	9.656±0.068
SY6	3.475±0.017	0.808±0.006	5.310±0.012	0.055±0.005	0.237±0.003	9.484±0.045
SA1	4.535±0.024	0.937±0.008	8.032±0.069	0.104±0.007	0.617±0.003	14.225±0.062
SA2	4.663±0.086	0.954±0.008	8.004±0.094	0.105±0.001	0.614±0.012	14.373±0.065
SA3	4.378±0.043	0.955±0.005	8.383±0.089	0.084±0.007	0.628±0.009	14.461±0.078
SA4	4.594±0.009	0.979±0.005	8.099±0.044	0.088±0.012	0.643±0.008	14.444±0.066
SA5	4.252±0.039	0.956±0.008	8.020±0.055	0.135±0.005	0.611±0.008	13.974±0.050
SA6	4.200±0.013	0.943±0.006	7.960±0.055	0.092±0.014	0.654±0.007	13.909±0.068
XJ1	3.831±0.018	0.886±0.006	6.504±0.067	0.078±0.001	0.512±0.007	11.811±0.045
XJ2	3.709±0.067	0.885±0.008	6.994±0.028	0.058±0.004	0.497±0.006	12.077±0.068
XJ3	3.810±0.043	0.895±0.013	6.476±0.050	0.057±0.003	0.515±0.009	11.752±0.048
XJ4	3.881±0.074	0.909±0.011	7.380±0.059	0.065±0.004	0.474±0.005	12.709±0.056
XJ5	3.871±0.064	0.887±0.011	6.619±0.094	0.082±0.007	0.479±0.008	11.937±0.059
XJ6	3.747±0.091	0.864±0.007	6.401±0.045	0.067±0.011	0.493±0.009	11.771±0.083

CD1	3.201±0.031	0.705±0.006	4.322±0.070	0.038±0.008	0.111±0.004	8.377±0.045
CD2	3.234±0.079	0.678±0.016	4.241±0.013	0.032±0.010	0.107±0.008	8.288±0.064
CD3	3.134±0.069	0.712±0.008	4.269±0.029	0.031±0.006	0.109±0.004	8.352±0.092
CD4	3.090±0.075	0.696±0.006	4.586±0.052	0.037±0.004	0.113±0.005	8.502±0.077
CD5	3.371±0.041	0.693±0.007	4.545±0.046	0.032±0.006	0.107±0.001	8.748±0.058
CD6	3.270±0.036	0.740±0.005	4.317±0.010	0.044±0.008	0.108±0.004	8.479±0.044
WZ1	3.649±0.091	0.829±0.005	5.837±0.052	0.071±0.005	0.329±0.008	10.715±0.081
WZ2	3.513±0.080	0.827±0.002	6.124±0.077	0.069±0.005	0.387±0.008	10.881±0.042
WZ3	3.688±0.040	0.815±0.009	6.242±0.056	0.054±0.008	0.317±0.008	11.116±0.069
WZ4	3.646±0.094	0.825±0.007	5.883±0.027	0.061±0.005	0.332±0.006	10.748±0.084
WZ5	3.715±0.086	0.830±0.004	6.224±0.073	0.054±0.008	0.357±0.007	11.150±0.063
WZ6	3.664±0.068	0.835±0.008	6.721±0.084	0.060±0.006	0.363±0.011	11.626±0.045
WR1	4.033±0.052	0.919±0.005	7.842±0.061	0.076±0.002	0.618±0.012	13.488±0.035
WR2	4.194±0.103	0.902±0.006	7.782±0.068	0.120±0.003	0.611±0.010	13.659±0.080
WR3	3.964±0.074	0.899±0.009	7.525±0.037	0.086±0.009	0.610±0.007	13.084±0.040
WR4	4.027±0.050	0.909±0.011	7.582±0.075	0.110±0.008	0.605±0.011	13.233±0.055
WR5	4.032±0.063	0.897±0.004	7.739±0.070	0.070±0.004	0.604±0.010	13.310±0.077
WR6	4.035±0.050	0.899±0.007	7.511±0.039	0.073±0.007	0.603±0.011	13.901±0.047
QY1	3.697±0.038	0.834±0.007	6.226±0.070	0.066±0.008	0.466±0.006	11.290±0.086
QY2	3.708±0.034	0.825±0.006	6.245±0.055	0.064±0.005	0.457±0.005	11.308±0.075
QY3	3.662±0.051	0.830±0.003	6.473±0.016	0.046±0.009	0.464±0.006	11.431±0.061
QY4	3.663±0.053	0.835±0.005	6.561±0.043	0.055±0.007	0.472±0.012	11.586±0.049
QY5	3.625±0.077	0.837±0.003	6.226±0.061	0.042±0.016	0.460±0.007	11.189±0.061
QY6	3.735±0.058	0.831±0.006	6.525±0.066	0.064±0.006	0.455±0.003	11.610±0.053

Table S2. ANOVA of the effects of cultivation region on the saikosaponin content

Factor	SSa	SSc	SSd	SSe	SSf
Cultivation region	–	–	–	–	–

" – " means significant difference at the level of $p < 0.05$.

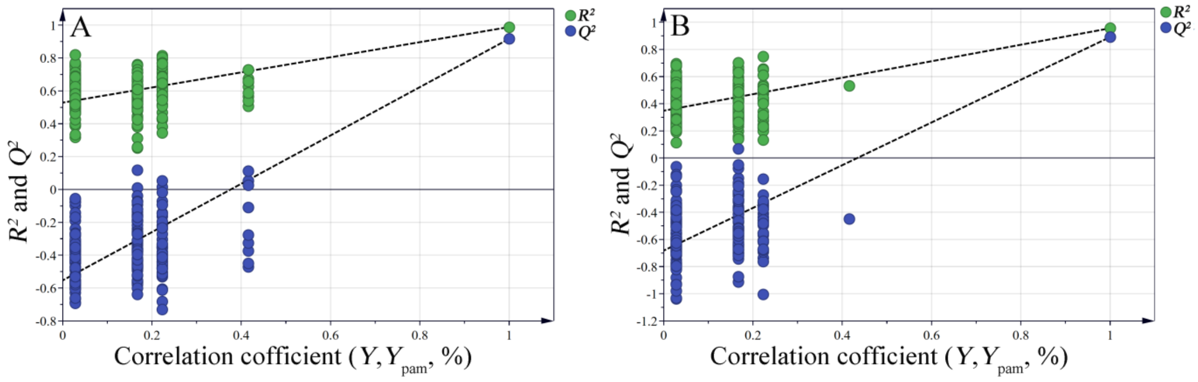

Figure S2. Permutation tests of PLS-DA models derived from the HPLC-MS datasets of saikosaponins (A) and the GC-MS datasets of volatile compounds (B) in the Radix Bupleuri samples
